# Design and Implementation of a Custom Built Optical Projection Tomography System

**DOI:** 10.1371/journal.pone.0073491

**Published:** 2013-09-04

**Authors:** Michael D. Wong, Jun Dazai, Johnathon R. Walls, Nicholas W. Gale, R. Mark Henkelman

**Affiliations:** 1 Department of Medical Biophysics, University of Toronto, Toronto, Ontario, Canada; 2 Mouse Imaging Centre (MICe), Hospital for Sick Children, Toronto, Ontario, Canada; 3 Regeneron Pharmaceuticals, Tarrytown, New York, United States of America; University of California, Irvine, United States of America

## Abstract

Optical projection tomography (OPT) is an imaging modality that has, in the last decade, answered numerous biological questions owing to its ability to view gene expression in 3 dimensions (3D) at high resolution for samples up to several cm^3^. This has increased demand for a cabinet OPT system, especially for mouse embryo phenotyping, for which OPT was primarily designed for. The Medical Research Council (MRC) Technology group (UK) released a commercial OPT system, constructed by Skyscan, called the Bioptonics OPT 3001 scanner that was installed in a limited number of locations. The Bioptonics system has been discontinued and currently there is no commercial OPT system available. Therefore, a few research institutions have built their own OPT system, choosing parts and a design specific to their biological applications. Some of these custom built OPT systems are preferred over the commercial Bioptonics system, as they provide improved performance based on stable translation and rotation stages and up to date CCD cameras coupled with objective lenses of high numerical aperture, increasing the resolution of the images. Here, we present a detailed description of a custom built OPT system that is robust and easy to build and install. Included is a hardware parts list, instructions for assembly, a description of the acquisition software and a free download site, and methods for calibration. The described OPT system can acquire a full 3D data set in 10 minutes at 6.7 micron isotropic resolution. The presented guide will hopefully increase adoption of OPT throughout the research community, for the OPT system described can be implemented by personnel with minimal expertise in optics or engineering who have access to a machine shop.

## Introduction

Ex-vivo 3D imaging at the mesoscopic scale (resolution = 2–50 µm) and with isotropic data acquisition is valuable for the observation and analysis of whole specimen and individual organ morphology. 3D imaging of morphology can give clues to physiological function; while aberrations in morphology can identify phenotypes when observed in genetically manipulated populations. Ex-vivo magnetic resonance imaging (MRI) has been successful in delineating anatomical differences in the mouse brain in models of autism and Huntington’s disease [1], [2] and describing cardiac malformations in the mouse embryo [3], [4] in knockout mice. Micro-CT can obviously detect malformations in bone structure [5] and with the development of iodine based contrast agents, micro-CT can produce images with surprisingly good soft tissue contrast, extending the potential applications to imaging the mouse embryo [6] and brain [7]. Episcopic Fluorescence Image Capture (EFIC) and High Resolution Episcopic Microscopy (HREM) are block-face imaging techniques that acquire superior resolution (∼1 µm) of sample autofluorescence [8] and sample attenuation of fluorescence of a resin-embedding media through eosin staining [9]. Both EFIC [10] and HREM [11] have been used extensively to analyze normal and aberrant mouse embryo phenotypes. Light sheet fluorescence microscopy (LSFM) or selective plane illumination microscopy (SPIM) employs a focused light sheet of excitation light which enables fast and high resolution imaging of small samples with minimal photobleaching; perfect for time lapse 3D images of development of live embryos [12]. Optical Coherence Tomography (OCT) is an interferometric technique to measure the optical scattering of tissue that can acquire high resolution (2–10 µm) 3D data sets at suitable depths (1–3 mm) for ex-vivo and in-vivo imaging of embryos early in development [13]–[15].

Optical projection tomography [16] has its own niche in the 3D imaging world as it can provide morphological information from tissue autofluorescence along with 3D visualization of gene expression through the use of fluorescent antibodies. It can image samples up to several cm^3^ in size and, depending on the sample size and objective lens used, OPT can resolve structures down to a few microns at isotropic resolution through use of a clearing agent termed BABB (1∶2 benzyl alcohol:benzyl benzoate). Projection data over the whole sample volume can be acquired within 10 minutes, which allows for studies with large sample sizes to be completed in a high-throughput fashion. The individual hardware components are readily available and the installation and software implementation is straightforward as well. This culminates in a stand alone cabinet OPT system with a general ease of operation. The combination of fast, high-resolution acquisition along with molecular specificity, ease of operation, and straightforward implementation makes OPT an attractive candidate to image ex-vivo whole mouse embryos and excised organs in 3D.

Over the last decade, OPT has been used to image a wide variety of organisms, tissues and biological systems. Numerous studies analyzing islet beta cell masses, the insulin producing cells in the pancreas, employed OPT to image, measure volumes, and depict locations of islet beta cell masses in concert with overall pancreatic morphology [17]–[20]. Mouse embryo vasculature has been imaged with OPT through PECAM-1 antibody staining to describe normal mouse vasculogenesis [21], and to view perturbations in vascular patterning due to genetic manipulations [22], [23]. Kidney ureter [24]–[26], human embryonic brain [27]–[30], vascular [31] and cardiac development [32] are presented in three-dimensions through OPT imaging to better describe these developmental processes spatially. Researchers are pushing OPT’s limitations in sample size with custom OPT implementations that can generate images of 3D structure of small samples such as those of single cells [33], [34] and large samples such as the adult mouse brain [35] and the adult mouse heart [36]. The combination of visualizing gene expression throughout the whole volume of mouse embryos and overall morphology with OPT proves to be essential for studies in neural [37], [38] and lung [39]–[42] development as well. OPT also has the potential to investigate in-vivo processes [43]. Modifications in hardware and the development of sophisticated software algorithms [44], [45] have made real-time visualization of internal structures possible, such as the skeletal and nervous systems of the zebrafish [46].

The majority of these studies were performed using a commercial OPT system distributed by the MRC Technology group named the Bioptonics 3001 OPT scanner. The remaining experiments were performed by custom in-house built OPT systems developed at research institutions with expertise in optics and engineering. The reason for this was two-fold: a limited number of the Bioptonics 3001 OPT scanners were produced and eventually this product was discontinued. The Bioptonics 3001 OPT scanner employed an uncooled CCD camera and an objective lens with a relatively small numerical aperture. The choice of the smaller numerical aperture ensured that the sample would be covered by an overly generous depth of field, but at a serious sacrifice in resolution. The company, Bioptonics, is no longer developing or selling new OPT systems and at present there is no OPT product that can be purchased by labs and institutes that need one.

Open access to design and operational protocols of imaging systems is essential for further development and its adoption in biological research. Open access initiatives have recently been published for both SPIM [47] and integrated microscopy systems [48]. Presented here are hardware and software components, along with assembly instructions, required to successfully build and install a fully functional optical projection tomography system. In addition, we describe algorithms to align the optics and moving stages and correct for deviations in the center of rotation. This reference will be essential for research institutes that would like to use OPT for their respective biological application but do not have the expertise to build a custom system from first principles.

## Materials and Methods

### System Parameters

The optical parameters of the presented OPT system are determined by the coupling of the 0.5× objective lens, 1.38× TV tube, and CCD. These parameters are listed in Table 1 for minimum and maximum magnification. The flexible zoom range is capable of imaging a sample as large as the adult mouse brain [35] and as miniscule as the E9.5 (Embryonic Day 9.5) embryo [49].

**Table 1:** Optical parameters of the described OPT system.

Mode	MAG.	N.A.	D.O.F. (mm)	F.O.V. (mm)	Pixel Size (µm)
Minimum Magnification	0.52	0.0094	6.2	13×13	12.8
Maximum Magnification	5.2	0.094	0.062	1.5×1.5	1.5

Optical parameters of the described OPT system are listed for both maximum and minimum magnification. MAG = magnification, N.A. = numerical aperture, D.O.F. = depth of field, F.O.V = field of view.

### Assembly Instructions

**Figure 1:**
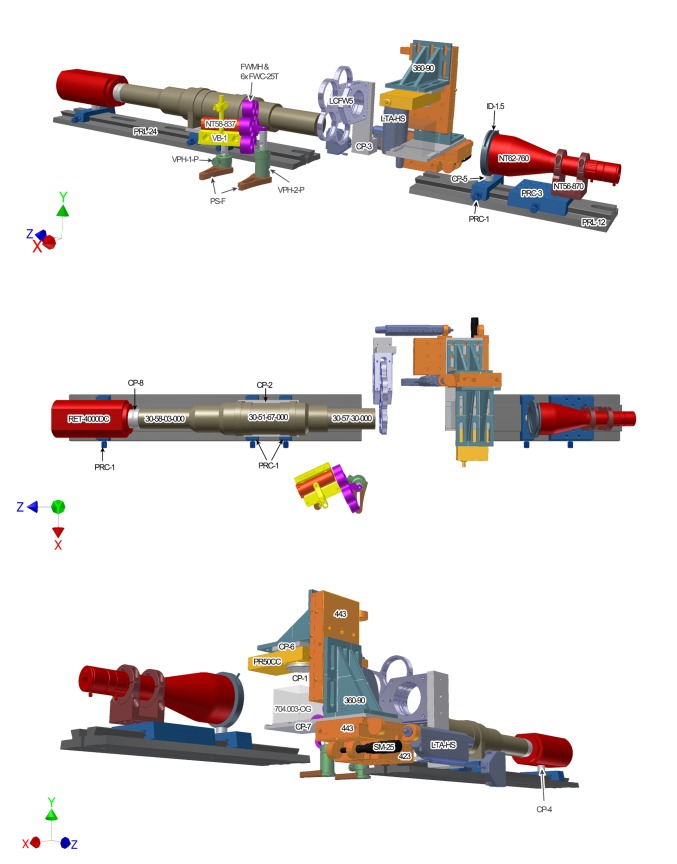
Diagram of custom-built OPT hardware set-up. OPT system set-up is presented in three different views to identify each hardware component and its relation to all other parts in the OPT system. The hardware parts are labeled in the view in which they are best displayed. The assembly of parts is described in the text and the parts list is available in Table 1.

Part numbers for the hardware are listed in Table 2 and a more detailed hardware parts list is available in Table S1. The arrangement of hardware components and the orientation of the Cartesian axes are demonstrated in Figure 1. Autodesk schematics for custom hardware are downloadable as supplementary material at www.mouseimaging.ca/technologies/opt.html. The minimum requirements for the OPT computer hardware includes a dual-core central processing unit (CPU) and a discrete graphics processing unit (GPU) with a modern version of the Windows operating system installed. The tested computer set-up was assembled with an Intel Xeon E5502 CPU, NVidia Quadro FX580 GPU and 4 GB of RAM running a 32-bit Windows XP Professional Service Pack 3 operating system. Labview by National Instruments and Matlab by Mathworks must be installed if the downloadable OPT controlling software and calibration software are to be used. The downloadable software were written and tested using Labview 2009 and Matlab 2009b, however, both pieces of software are compatible with, and were recently tested on, Labview 2012 and Matlab R2012a. The Matlab image processing toolbox and the National Instruments Vision Development module must also be installed to use the supplied OPT software.

**Table 2:** OPT hardware parts list.

Element	Vendor	Description	Part #	Quantity
**Breadboard**	Newport	Optical Breadboard	RG-25-4-ML	1
**Microscope**	Qioptiq	“C” Mount Coupler	33-02-00-000	1
	Qioptiq	1.38x TV tube	30-58-03-000	1
	Qioptiq	Junction box	30-17-74-000	1
	Qioptiq	Wall transformer	30-17-31-000	1
	Qioptiq	Upper core zoom w/stepper motor	30-51-67-000	1
	Qioptiq	0.5X Objective	30-57-30-000	1
	Qioptiq	Stepper Motor Drive	30-17-78-000	1
	Qioptiq	Connector DB25	301730-802	1
	Qioptiq	Connector DB9	301730-803	1
**CCD**	Qimaging	Retiga 4000DC	RET-4000DC-R-F-M-12-C	1
	Custom	Custom CCD-to-microscope adapter	CP-9	1
	Custom	Height Spacer	CP-4	1
**Cuvette**	Hellma	50 mm Light path large cell	704.003-OG	1
	Custom	Cuvette holder	CP-8	1
**Stages**	Newport	Linear stage, 2.0 inch travel	443	2
	Newport	Linear stage, 1.0 inch travel	423	1
	Newport	Rotation stage	PR50CC	1
	Newport	90° Angle bracket	360-90	2
	Newport	Vernier micrometer, 25 mm travel	SM-25	1
	Newport	Power Supply	SMC-PS80	1
	Newport	Motorized actuator, 50 mm travel	LTA-HS	2
	Newport	Single-axis motion controller/driver	SMC100CC	3
	Newport	Rail Carrier	prc-3	1
	Newport	Rail Carrier	prc-1	4
	Newport	24" Precision Dovetail Rail	prl-24	1
	Newport	12" Precision Dovetail Rail	PRL-12	1
	Newport	USB Interface	SMC-USB	1
	Custom	Steel disk mount for rotation stage	CP-1	1
	Custom	Adapter plate: microscope-rail guides	CP-2	1
	Custom	Adapter plate:PR50CC-to-360-90	CP-6	1
**Illumination (WH)**	Edmund Optics	Telecentric Backlight Illuminator	NT62-760	1
	Edmund Optics	Fiber Optic Light Guide Adapter	NT38-944	1
	Edmund Optics	Flexible Fiber Optic Light Guide	NT42-346	1
	Edmund Optics	Silver Series Mounting Clamp	NT56-870	1
	Edmund Optics	115V, MI-150 Fiber Optic Illuminator	NT59-235	1
	Newport	Iris Diaphragm	ID-1.5	1
	Newport	Post Mounting Ring	LT10-PR	1
	Custom	Height spacer for iris	CP-5	1
	Custom	Height spacer for telecentric lens	CP-7	1
**Illumination (UV)**	EXFO	X-Cite exacte system with lamp	P010-00230R	1
	Edmund Optics	Focusing Assembly	NT58-837	1
	Semrock	425/30 BrightLine bandpass filter	FF01-425/30-25	1
	Semrock	473 RazorEdge long-pass Filter	LP02-473RU-50.8-D	1
	Newport	Filter Wheel Hub	FWMH	1
	Newport	Filter Carrier	FWC-25T	6
	Thorlabs	60 mm Cage Filter Wheel	LCFW5	1
	Newport	Optical Post Holder, 1.9 inch	VPH-1-P	1
	Newport	Optical Post Holder, 2.19 inch	VPH-2-P	1
	Newport	Clamping Fork	PS-F	2
	Newport	Optical Mounting Post, 0.5 inch	SP-1.5	2
	Newport	V-Block	VB-1	1
	Custom	Emission filter wheel mount	CP-3	1

All parts needed for the presented OPT system are listed here, both commercially available and custom-made. Illumination assembly is separated for hardware parts required for white light (WH) and ultraviolent (UV) illumination. A more descriptive parts lists along with optional system additions are included in Table S1.

#### Step 1: Base

Attach the long optical rail (PRL-24) to the optical breadboard (RG-25-4-ML) along its longest dimension (z-axis). Attach the shorter optical rail (PRL-12) on the breadboard coaxially to the longer rail on the z-axis. The distance between the two optical rails should be approximately 30 cm.

#### Step 2: Translation stages

Attach a stage (423) with a Vernier micrometer (SM-25) to the optical table such that the stage axis of movement is along the x-axis, perpendicular to the optical rails. This will be act as fine adjustment along the x-axis to place the center of rotation in the middle of the field of view (FOV) of the camera. Attach another stage (443) with a motorized actuator (LTA-HS) on top of the 423 stage such that it actuates along the z-axis, the same axis as the optical rail. This will act as the focus motor in the OPT system, working to bring the sample into and out of the focal plane. Place the custom cuvette holder (CP-7) on top of the 443 stage. Using screws, secure a 90° angle bracket (360–90) and the cuvette holder to the 443 stage. Attach another stage (443) with a motorized actuator (LTA-HS) to the 90**°** angle bracket so that the stage movement will be along the y-axis. This stage will act as fine adjustment for the sample along the y-axis so it can be centered into the FOV of the camera. Lastly, attach another angle bracket (360–90) to the vertical 443 stage. This bracket will be used to mount the rotation stage.

#### Step 3: Rotation stage

Attach the custom magnetic disc (CP-1) and the custom rotation stage interface plate (CP-6) to the rotation stage (PR50CC). Next, attach the rotation stage assembly to the angle bracket so that the magnetic disc is facing downwards (along y-axis).

#### Step 4: Microscope

First, attach the custom microscope interface plate (CP-2) to two rail carriers (PRC-1). Then, attach the microscope interface plate to the upper core of the microscope (30-51-67-000). Place the rail carriers on the long optical rail and then attach the objective lens (30-57-30-000) and TV tube (30-58-03-000) to the upper core of the microscope.

#### Step 5: CCD

To align the heights of the CCD (RET-4000DC-R-F-M-12-C) and microscope, place the custom spacer (CP-4) between the CCD and rail carrier (PRC-1) and screw the three components together. This requires the underside of the center hole on the rail carrier to be counterbored to accommodate the screw. Screw the custom CCD-to-microscope adapter (CP-9) onto the CCD. Place the rail carrier for the CCD onto the long optical rail and slide the CCD assembly onto the microscope and fasten the setscrews.

#### Step 6: White light and iris diaphragm

Attach the telecentric backlight illuminator (NT62-760) to the clamp (NT57-870) and mount this assembly onto a rail carrier (PRC-3) using a shim washer between the clamp and rail carrier to elevate the illuminator to the same height as the microscope and CCD. Mount this new illuminator assembly to the short optical rail, facing the microscope. Mount the iris diaphragm (ID-1.5) to a rail carrier (PRC-1) with a custom spacer (CP-5) between these two components to elevate and align the center of the iris to the center of the microscope assembly along the optical axis (z-axis). Place the iris diaphragm assembly on the short optical rail just in front of the telecentric illuminator. The adjustable iris is needed when using the microscope at high magnifications, to minimize the contributions of internal reflections within the illuminator and, therefore, produce a homogeneous bright-field over the FOV of the camera. Lastly, (not shown in Figure 1) attach the white light source (NT59-235) to the back-end of the illuminator by fiber-optic cable (NT42346) using the appropriate adapter (NT38-944).

#### Step 7: Emission filter wheel

Attach the emission filter wheel (LCFW5) to the custom filter wheel mount (CP-3). Position and screw down the filter wheel assembly onto the breadboard such that the filters are positioned in front of the objective lens and that the objective does not interfere with the rotation of the filter wheel.

#### Step 8: Excitation focusing lens and filter wheel

Mount the V-Block (VB-1) to an optical mounting post (SP-1.5) and insert the post into a 1 inch post holder (VPH-1-P). Mount the excitation filter wheel assembly (6x FWC-25T, FWMH) to an optical mounting post (SP-1.5) and insert the post into a 2 inch post holder (VPH-2-P). Position and clamp the V-Block and excitation filter wheel assemblies to the breadboard using clamping forks (PS-F). Clamp the excitation focusing lens (NT58-837) to the V-Block. Insert the UV light source (P010-00230R) into the focusing lens through fiber optic (not shown in Figure 1).

#### Step 9: Initialization of hardware components on the personal computer

Connect each actuator cable to its own controller box (SMC100CC). Using the provided manufacturers software and instructions, initialize each actuator on your computer, each with their own respective channel. Test the functionality of each actuator using the manufacturers software before attempting to use them with the provided Labview OPT software. Connect the CCD to the computer and install the manufacturer supplied device drivers and software. Again, make sure the camera is operational via the manufacturer software before using the supplied Labview program. Lastly, connect the microscope controller to the computer and initialize the zoom mechanism using the supplied drivers and software. Once the hardware components are recognized and functional through PC connection, the provided Labview software template can be used for the subsequent presented alignment and image acquisition steps.

### Alignment Procedure

The following is a description of a method to ensure proper optical alignment of the aforementioned hardware components which is essential for successful OPT image acquisition. One can use the software supplied by the microscope, camera, and stage actuator manufacturers in tandem to execute this alignment process. Equivalently, the downloadable Labview software integrates all these controllers together, which may be the most robust method in carrying out the following procedure. A downloadable Matlab script can be used to make the calibration process more user friendly as well. Code for operational and calibration software can also be found at www.mouseimaging.ca/technologies/opt.html.

A phantom is required for the outlined alignment process. For example, a 1 mm ball bearing embedded in low-melting point agarose cleared with BABB (2∶1 benzyl benzoate, benzyl alcohol solution).

#### Step 1: Positioning the optics

The microscope-CCD assembly should be positioned on the long optical rail such that the objective lens is its working distance away (16cm) from the center of the focusing stage’s travel range (i.e. 25 mm). The easiest way to approach this is to suspend an object from the rotation stage, set the focusing actuator to 25 mm, and move the microscope-CCD assembly along the z-axis of the optical rail until the object is in focus.

#### Step 2: Align CCD and microscope

Turn on the white light source. At minimum zoom, suspend the ball-bearing phantom from the rotation stage and use the height displacement actuator (y-direction) to place the ball bearing at the center of the FOV of the CCD. Increase the zoom level towards maximum zoom. If aligned correctly, the centroid of the ball bearing would remain in the center of the FOV. If the centroid moves during zoom action, adjust the screws on the camera-microscope mount to attain columnarity.

#### Step 3: Align the stage assembly with the microscope

With the ball bearing at the center of the CCD FOV, move the focus actuator (z- direction). Again, if the stage assembly is perfectly aligned with the microscope-CCD assembly the centroid of the ball bearing should not move with the focus motor. If the stages are not aligned with the optics, rotate the stage assembly in the XZ plane until the focus motor is parallel to the microscope. Re-test and rotate again if necessary.

At minimum zoom, center the ball bearing on the center vertical of the FOV and place it at the bottom of the FOV with the height actuator. Raise the ball bearing in the y-direction until the bead reaches the top of the FOV. If the ball bearing deviates from the center vertical, shim the stage assembly to rotate the stage assembly in the XY plane until this test is passed.

**Figure 2:**
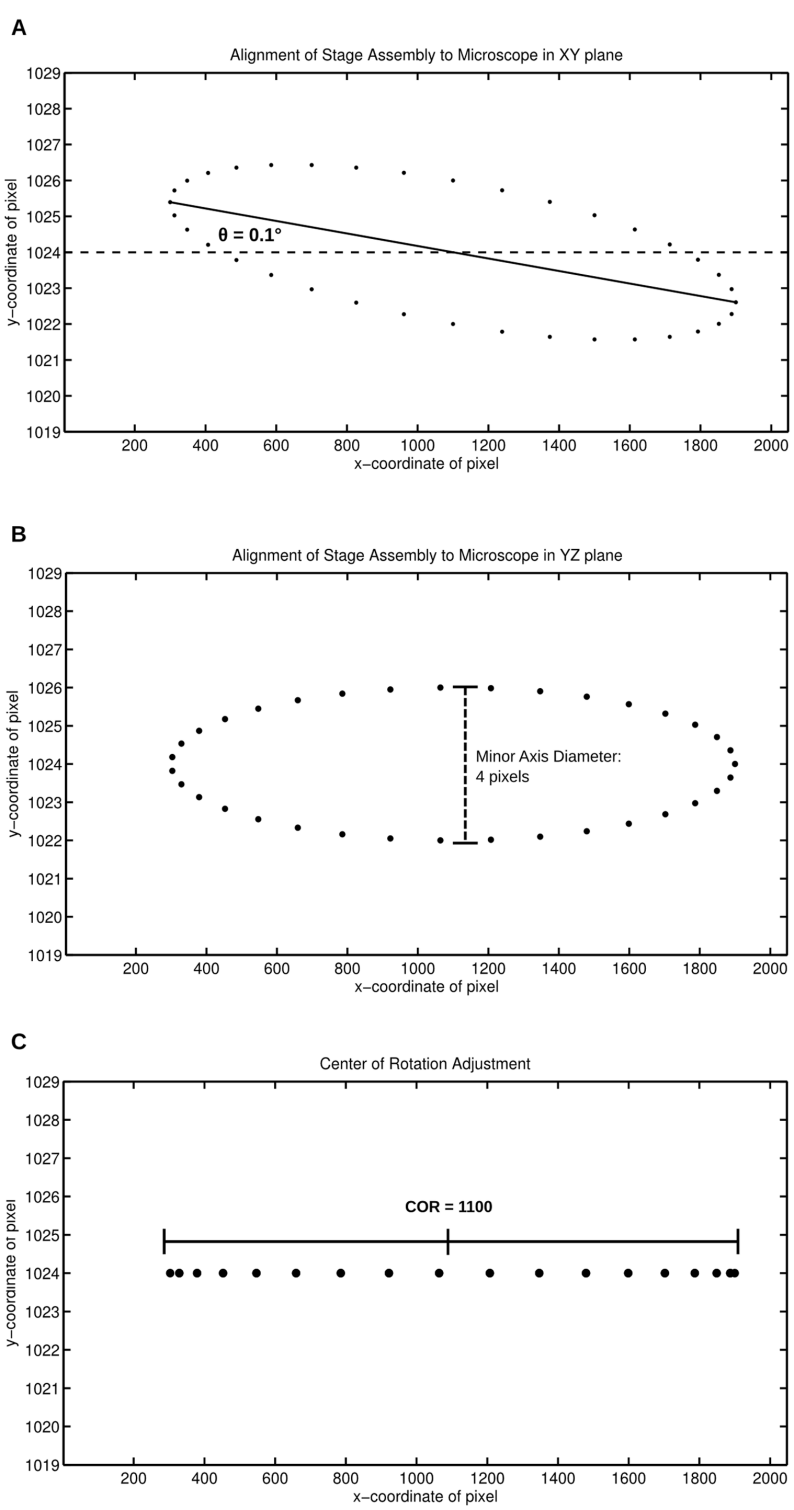
Alignment of the stage assembly with the OPT microscope carving out an ellipse using a bead phantom. In this example, the stage assembly is rotated in both the XY and YZ planes with respect to the microscope and the center of rotation is currently at pixel 1100. (A) To align the stage assembly with the microscope in the XY plane, the angle (θ) between the x-axis and the major axis of the ellipse should be reduced from 0.1 to less than 0.01. (B) To align the stage assembly with the microscope in the YZ plane, the diameter of the minor axis should be reduced from 4 pixels to less than 1.5 pixels. (C) To move the center or rotation to the center of the CCD, move the stage assembly such that the center of the ellipse is positioned at the center pixel of the field-of-view (i.e. 1024).

At a medium zoom setting, set the ball bearing away from the center of rotation such that throughout a 360 degree rotation, the ball bearing does not escape the FOV. Acquire an image of the ball bearing at 10 degree steps throughout one revolution. In Matlab or equivalent graphing program, trace the centroid of the ball bearing throughout the 360 degree rotation. The path should appear as an ellipse (Figure 2a).

The misalignment due to a rotation in the XY plane can be determined by calculating the angle between the x-axis and major axis of the ellipse (Figure 2a). Optimally, this angle, θ, should be zero. Shim between the custom cuvette holder (CP-7) and the focus stage (443) to rotate the stage in the XY plane, such that θ is less than +/−0.01 degrees.

Once the misalignment due to the rotation θ in the XY plane is reduced to zero, the extent of which the system is misaligned due to a rotation in the YZ plane is proportional to the diameter of the minor axis of the ellipse (Figure 2b). To correct this, the 360-90 bracket that holds the rotation stage can be re-positioned on the vertical stage (443) by re-adjusting the screw locations, resulting in a rotation in the YZ plane. Redraw the ellipse and repeat the aforementioned hardware adjustments until the diameter of the minor axis is less than 1.5 pixels (Figure 2c).

The center of rotation (COR) of the system can be measured as the x-coordinate of the center of the ellipse (Figure 2c). Use the vernier micrometer to move the stage assembly in the x-direction until this center of rotation value is at the center of the CCD.

### Acquisition Software (Labview)

**Figure 3:**
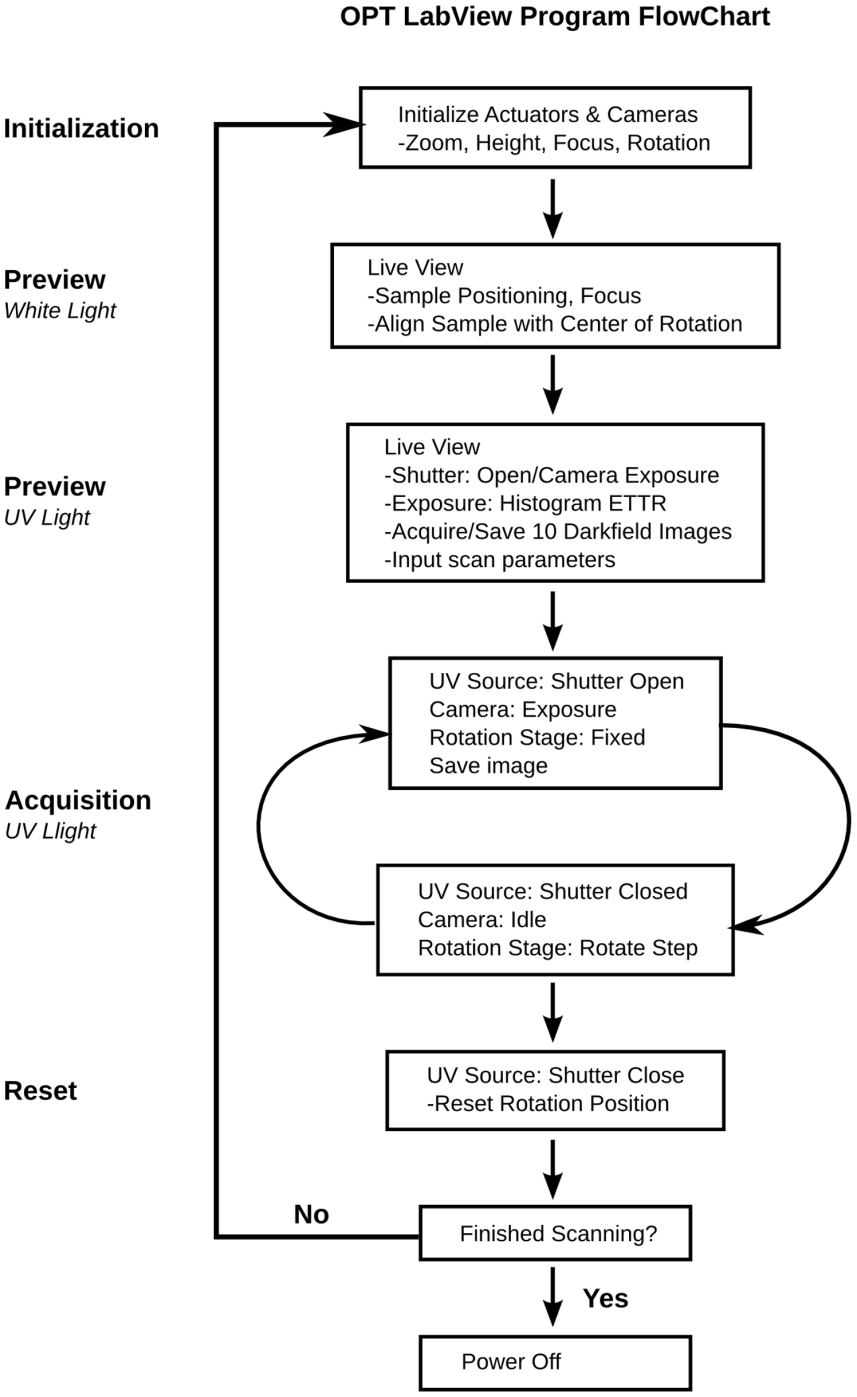
Flow chart of the Labview OPT software.

A copy of our custom Labview program for image acquisition and Matlab code for the conversion of the raw binary image files to compatible tiff files for the computer tomography reconstruction software (Skyscan Nrecon) can be found at (www.mouseimaging.ca/technologies/opt.html). Brief descriptions of each component of the software are presented below. A flow chart of the acquisition software is illustrated in Figure 3.

#### Initialization

The camera and zoom motor must be powered on, before the Labview program is initiated. Once initialized, the three actuators, focus motor, rotation motor, and height motor are homed and reset to their default positions.

#### Live preview (White light)

The white light source is powered on to properly position the sample within the field-of-view of the camera. CCD camera settings such as exposure time, electronic gain, image size, and binning can all be adjusted in the live view. The sample is placed in the OPT system and is suspended from the rotation actuator stage with magnetic contacts. The sample is then lowered into the BABB filled cuvette, by way of the height actuator, until it is fully immersed and within the field of view of the CCD. The sample is centered by fine adjustments to the height actuator along with manual fine adjustments in the x-direction with a set of forceps. To ensure that the sample is centered on the axis of rotation, the sample is rotated 90 degrees, repositioned to the center of the CCD in the x-direction, and rotated another 90 degrees. This is done iteratively until the sample is observed to be rotating on the motors center of rotation. The focus motor is then adjusted until the sample is visually ‘in-focus’.

#### Live preview (UV Light)

The white light source is powered off and the UV source powered on when imaging in emission mode. The appropriate excitation and emission filters are placed in front of the excitation light and objective respectively by way of a filter wheel so that the appropriate wavelength can be used for a chosen fluorophore. The focus motor is finely re-adjusted because of the different wavelength of light. The exposure time for the CCD camera is adjusted so that within the live histogram, the image is Exposed To The Right (ETTR) so that the brightest pixels are close but not more than the ceiling of the 12-bit CCD. This is to maximize the dynamic range so the images will utilize all 12-bits available in the camera. This should be checked at different projection angles as the acquired cumulative signal varies with the orientation of the sample. Once exposure is finalized, the sample is lifted out of the FOV and 10 dark-field images are acquired, which will be later averaged and subtracted from each projection. The sample is then lowered to the exact position it was before and then the scan parameters such as angular step size and number of averages can be updated.

#### Acquisition (UV Light)

At the initialization of the scan, the system enters a loop in which a projection at each angular step is acquired, saved to disk, and then the rotation motor rotates the sample at the designated angular step. This is repeated for the number of projections chosen by the user (360/angular step size). Of special note, the CCD can also be used to trigger the shutter of the UV light source. This will minimize photobleaching; eliminating UV illumination of the sample while the image is being saved to disk as well as while it is rotating through each angular step. The Labview program displays each projection that is acquired, providing a ‘slowly’ refreshing live view. This will allow the user to determine if the sample has shifted or has fallen during the scan acquisition as a result of inadequate mounting.

#### Reset

At the end of the acquisition, the rotation motor has revolved the sample back to its initial so that another scan can be acquired using another channel easily and in the same space. In addition, the shutter of the UV light source is closed after a full acquisition to minimize photobleaching. At this point, another scan can be initiated or the Labview program can be powered off along with the hardware components.

#### Preparation for image reconstruction

The raw data output from the scan must be converted into uncompressed TIFF images before it can be read by the Skyscan Nrecon software. In addition, projection image averaging and the subtraction of background images must be performed as well. The supplied Matlab code averages the raw data and subtracts each projection image from an average of 10 background images before converting to TIFF. These operations are executed post image acquisition in Labview to minimize the processes performed during a scan and, therefore, minimizing scan time and photobleaching. The recon software, Skyscan Nrecon, is identical to that shipped with the commercial Bioptonics scanner and is straightforward to use.

### System Characterization

**Figure 4:**
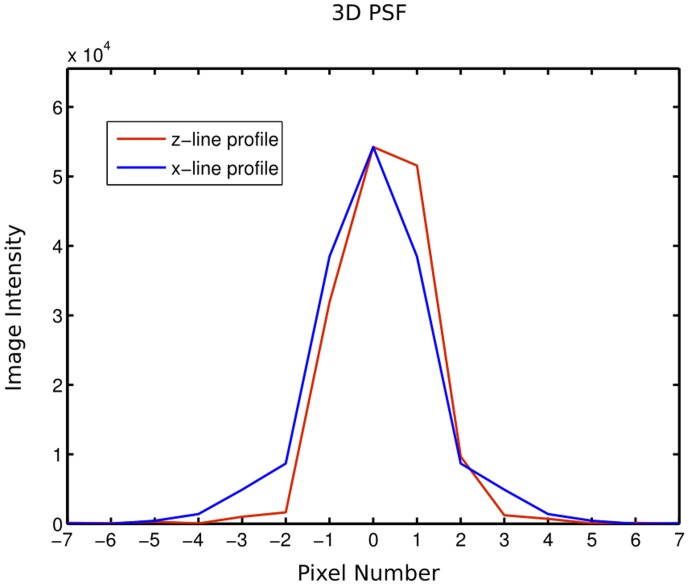
3 dimensional point spread function (PSF) of the described OPT system. Line profiles of the image intensity through the center of a fluorescent bead along the x (blue) and z (red) axes discretized by pixel number. The full width at half maximum of these line profiles is demonstrative of the lateral and axial resolution of the system (6.77 and 6.72 microns respectively).

An investigation of the resolution of the system is measured empirically by measuring the point spread function (PSF) in 3D by imaging a fluorescent bead with a diameter less than that of the system resolution. This measurement was taken at a magnification of 2.871, which corresponds to a stepper count value of 12307 in the Labview software. This magnification corresponds to an image pixel size of 2.45 microns. A line profile of image intensity through the center of the bead along the x and z axes is presented in Figure 4 to determine the lateral and axial resolution respectively. The full width at half maximum is 2.76 and 2.74 pixels for the x and z line profiles respectively. As a result, the lateral and axial resolution of the presented OPT system is 6.77 and 6.72 microns. The resolution at other magnifications can be calculated from these measured values and can be used to determine if an OPT system built using this guide is performing as expected optically.

## Results and Discussion

**Figure 5:**
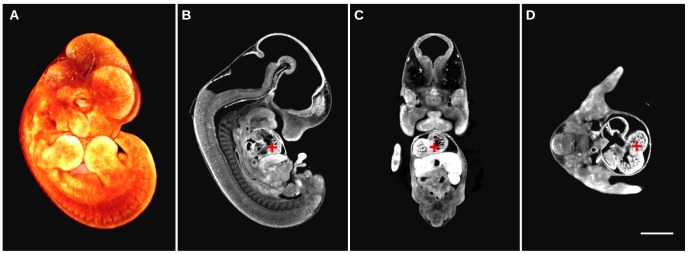
E12.5 mouse embryo autofluorescence image acquired with the presented custom OPT system. (A) 3D textured rendering of the whole volume of the mouse embryo is illustrated in orange. Digital sections of the same mouse embryo are illustrated in gray-scale demonstrating autofluorescence anatomy data in sagittal (B), coronal (C), and axial planes (D). An equivalent location in anatomy is shown by the location of the red cross-hair. The scale bar is 2 mm.

**Figure 6:**
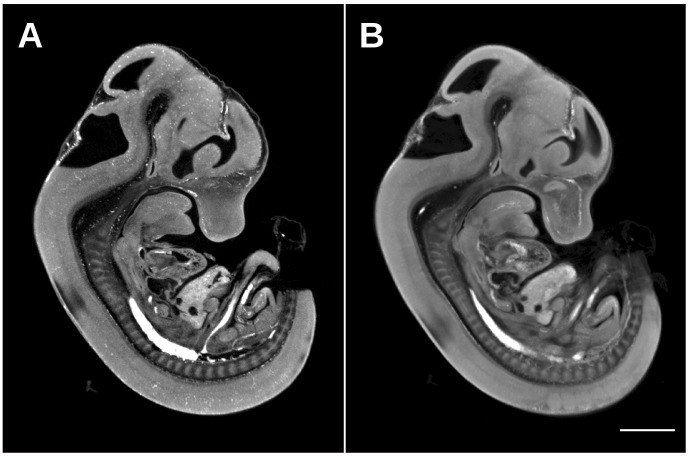
E12.5 mouse embryo autofluorescence image acquired by the presented custom OPT and the commercial Bioptonics 3001 OPT Scanner. Similarly positioned sagittal sections through an E12.5 mouse embryo acquired by the custom OPT (A) and the Bioptonics system (B). The higher resolution produced by the custom scanner is visually evident through sharper and more defined edges as well as the observation of individual blood pooling in the vasculature. The scale bar is 2 mm.

Presented here is a detailed description of how to assemble, install, and use a custom optical projection tomography system that is capable of generating 3-dimensional data sets like Figure 5. The value of capturing a 3D image demonstrating morphology over the whole volume of the sample is evident in Figure 5, where an autofluorescence image of an E12.5 C57Bl/6 mouse embryo can be sliced digitally and therefore viewed at any arbitrary orientation such as sagittal, axial, and coronal to observe locations of interest (Figure 5b,c,d). In addition, the benefit of a custom OPT with a better CCD camera and an objective lens with a larger numerical aperture over the commercial Bioptonics OPT 3001 Scanner is demonstrated in Figure 6. The higher resolution of the custom built OPT system is visually evident in Figure 6a where the overall morphology of the E12.5 mouse embryo is sharper and more defined compared to a similar E12.5 mouse embryo autofluorescence image acquired with the Bioptonics 3001 OPT scanner (Figure 6b). This difference in resolution is most evident in the ability to resolve punctate individual blood pooling in the mouse embryo vasculature in Figure 6a versus Figure 6b. Both 3D data sets were acquired at the same magnification and the generated images have an equivalent pixel size. In addition, both 3d data sets run through the same Skyscan recon software with the same parameters.

The data output of the presented custom OPT system can also be manipulated for resolution improvement using published methods [50]. An application where the enhanced resolution of the custom-built system and the resolution improvement detailed by Walls et. al [50] is needed is early vasculogenesis and vascular patterning in the early mouse embryo. These early blood vessels are approximately 5–10 microns in diameter, which require a resolution that the Bioptonics 3001 OPT scanner cannot achieve. Therefore, this new scanner with FDR and deconvolution can give the requisite <3 micron resolution.

The presented system is capable of acquiring a full data set of 1200 projections in one channel within approximately 10 minutes with a typical exposure time of 500 ms. This is comparatively very fast considering that the generated data set can achieve a pixel size of 3–5 microns over the volume of a whole mouse embryo. However, the largest gain in throughput would be an automated system in which samples can be imaged sequentially without any user intervention. The presented implementation of an OPT system is a starting point to such a goal, where the Labview program could be modified to position, focus and scan a sample without user input. The real challenge is mounting samples onto rotation stages one after the other to be scanned, as the samples must be stored in BABB before, during, and after image acquisition. The successful addition of robotics to mount these samples onto the OPT system could result in a scan rate of approximately 120 samples scan in a 24 hour day. This would be incredibly useful in large-scale applications such as the International Mouse Phenotyping Consortium (http://www.mousephenotype.org) from which approximately 30% of 23,000 mouse lines will require mouse embryo phenotyping. 3D imaging has been accepted as a primary screen for embryonic lethal mouse lines and OPT is a leading imaging option for E9.5 mouse embryo screening [51]. The system described here can be installed in production centers internationally as well as individual research labs at relatively low cost and does not require much physical space or specialized expertise by users. The presented OPT system costs approximately $80,000 to build in hardware parts alone; a relatively low cost compared to commercial high-resolution 3D ex-vivo imaging systems. It is hoped that this paper will help to eliminate the barrier to entry and enable the wider spread use of OPT throughout the research community.

## Supporting Information

Table S1 Detailed OPT hardware parts list. A more detailed description of each OPT hardware part than the information provided in Table 2.
(XLS)
